# Laparoscopic management of mesenteric cyst: a case report

**DOI:** 10.1186/1757-1626-2-132

**Published:** 2009-02-08

**Authors:** Theodoros D Theodoridis, Leonidas Zepiridis, Dimitrios Athanatos, Filippos Tzevelekis, Diamantis Kellartzis, John N Bontis

**Affiliations:** 1 First Department of Obstetrics and Gynaecology, Aristotle University of Thessaloniki, 'Papageorgiou' Hospital, Thessaloniki, Greece

## Abstract

Mesenteric cysts are rare intra-abdominal lesions with variable clinical symptoms and signs that make pre-operative diagnosis difficult. Optimal treatment is surgical excision of the cyst with laparotomy or laparoscopy. We present a case of mesenteric cyst that was misdiagnosed as para-ovarian cyst and managed laparoscopically by gynaecologists.

## Introduction

Mesenteric cysts are rare intra-abdominal tumors with prevalence 1:100.000 in adults and 1:20.000 in children [1,2]. They are usually benign and asymptomatic, but occasionally they present with various, non-specific symptoms. Due to the rarity of this entity and the lack of specific symptoms, correct pre-operative diagnosis is difficult. Complete surgical excision is the treatment of choice. This can be acomplished by laparotomy or by minimally invasive surgery.

We present a case of mesenteric cyst that was misdiagnosed as para-ovarian cyst and managed successfully laparoscopically.

## Case report

A 30 years old, G0P0, Caucasian, housewife, refer red to our department for surgical management of a left-sided para-ovarian cyst. Her only complaint was a vague, non-specific lower abdominal pain for the previous 6 months. She had an appendicectomy at the age of 12 but the rest of her medical and family history were unremarkable.

**Figure 1:**
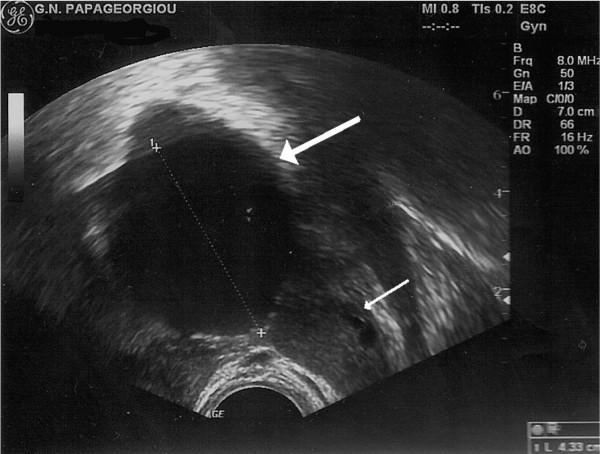
**Thick arrow: cystic structure missdiagnosed as para-ovarian cyst in ultrasound**. Thin arrow: left ovary.

Clinical examination was also unremarkable and her blood tests were normal. Bimanual gynaecological examination revealed a palpable, motile tumor in the left adnexae. Trans-vaginal ultrasound revealed a 4.33 × 4.84 × 4.52 cm thin-walled, simple, unilocular cyst, located next to the left ovary (Fig. 1). A para-ovarian cyst was the most likely diagnosis, hence a laparoscopic removal was proposed.

At laparoscopy a simple cyst was seen lying inferior and lateral to the aortic bifurcation, arising from the mesenterium of the descending colon, in contact with left ovary. During exploration the cyst was found movable, attached only posteriorly to the mesenterium. The cyst was carefully disected with the assistance of ultracision harmonic scalpel (Ethicon, Endo-Surgery, LLC) and removed intact. Thorough examination of the integrity of the bowel and the mesenteric vessels was followed. The specimen was placed in an endobag, aspirated and extracted from the peritoneal cavity through the 10 mm suprapubic port. Histopathological examination revealed a simple, benign mesothelial cyst of the mesenterium.

The patient's post-operative period was uneventful and she was discharged free of symptoms the following day. One year after the surgery she remains completely asymptomatic.

## Discussion

Mesenteric cysts are quite rare intra-abdominal lesions. Italian anatomist Benevenni first described this entity performing an autopsy in an 8 years old boy in 1507 [3].

In most of the cases the cysts are located in the mesenterium of small intestine, but they can also be found in the descending colon and rectum [4]. Their histopathological classification is designated by the cell type of the inner cyst wall layer, so mesenteric cysts can be of lymphatic, mesothelial, enteric, urogenital origin, or nonpancreatic pseudocysts [1,2]. Fortunately they behave mostly as benign tumors, while malignancy accounts for 3% of the cases, arising gradually or de novo [5].

Mesenteric cysts size ranges between a few centimeters and 10 cm [6]. Usually they do not cause specific symptoms. Clinical presentations include common symptoms of the gastrointestinal tract and rarely they can be complicated with rupture, torsion or intestinal obstruction causing acute and intense symptoms.

Correct pre-operative diagnosis is quite difficult due to the rarity of the entity and the lack of specific symptoms and signs. It can be achieved when the phycisian is alerted concerning this condition and includes it in the differential diagnosis of intra-peritoneal cysts. Clinical imaging (ultrasound, CT scan or Magnetic Resonance Imaging-MRI) [6] may aid in the correct diagnosis. Careful interpretation of images is essential. Despite our experience this cystic structure was missdiagnosed as para-ovarian in origin, hence we believe that CT scan should be considered more often in preoperative management.

The treatment of choice is complete surgical excision of the cyst. This can be done either by laparotomy or laparoscopy [7,8]. The decision regarding the surgical approach depends on the size of the cyst, its location in the abdominal cavity and eventually the level of surgeon's experience in minimal access surgery. In our case the procedure was performed by experienced Gynaecological endoscopists.

## Conclusion

Mesenteric cysts, although quite rare tumors of the mesenterium, must always be considered in differential diagnosis of pelvic cystic lesions. Laparoscopic enucleation of mesenteric cysts is feasible and should be considered as the treatment of choice.

## Consent

Written informed consent was obtained from patient – in her native language – for publication of this case report and accompanying image. Copy of the written consent is available for review by the Editor-in-Chief of this journal.

## Competing interests

The authors declare that they have no competing interests.

## Authors' contributions

TDT and LZ conceived the study and participated in patient management and surgery, acquisition of data, interpretation of data, and were major contributors in writing the manuscript. DA participated in patient management, acquisition of data, and drafting of the manuscript. FT and DK revised critically the manuscript adding substantial intellectual content. JB coordinated the study and patient management and revised critically the manuscript. All authors have read and approved the final manuscript. The manuscript is not under consideration and has not been published by another journal.
